# Expression of a rK39 homologue from an Iranian *Leishmania infantum* isolate in *Leishmania tarentolae* for serodiagnosis of visceral leishmaniasis

**DOI:** 10.1186/s13071-019-3839-3

**Published:** 2019-12-18

**Authors:** Zahra Rezaei, Nick Van Reet, Gholamreza Pouladfar, Vera Kühne, Amin Ramezani, Bahador Sarkari, Bahman Pourabbas, Philippe Büscher

**Affiliations:** 1 Department of Parasitology and Mycology, School of Medicine, Shiraz University of Medical Sciences, Shiraz, Iran; 2 Professor Alborzi Clinical Microbiology Research Center, Shiraz University of Medical Sciences, Shiraz, Iran; 3 Department of Biomedical Sciences, Institute of Tropical Medicine, Antwerp, Belgium; 4 Institute for Cancer Research, Shiraz University of Medical Sciences, Shiraz, Iran; 5 Basic Sciences in Infectious Diseases Research Center, Shiraz University of Medical Sciences, Shiraz, Iran

**Keywords:** rK39, *Leishmania*, Serodiagnosis, Visceral leishmaniasis, Eukaryotic expression

## Abstract

**Background:**

Kinesin-related gene diversity among strains and species of *Leishmania* may impact the sensitivity and specificity of serodiagnostic tests for visceral leishmaniasis (VL).

**Methods:**

In this study, we report on the recombinant expression of this novel Iranian *Leishmania infantum* (MCAN14/47) homologue of rK39 (*Li*-rK39), in *L. tarentolae*. The diagnostic potential of the *Li-*rK39 antigen was evaluated in an ELISA, using sera from 100 VL patients, 190 healthy endemic controls, 46 non-endemic healthy controls and 47 patients with other infections.

**Results:**

The results showed a sensitivity of 96% and a specificity of 93.8%. A commercial rK39 immunochromatographic test (ICT) was 90% sensitive and 100% specific on the same cohort.

**Conclusions:**

Here, we show that the K39 gene from an Iranian *L. infantum* isolate is heterozygous as compared to the sequence of the Brazilian *L. infantum* (former *L. chagasi*), whose antigen is incorporated in most rK39-based immunochromatographic tests. Therefore, *Li-*rK39 has the potential to be used as an alternative for VL diagnosis in Iran.
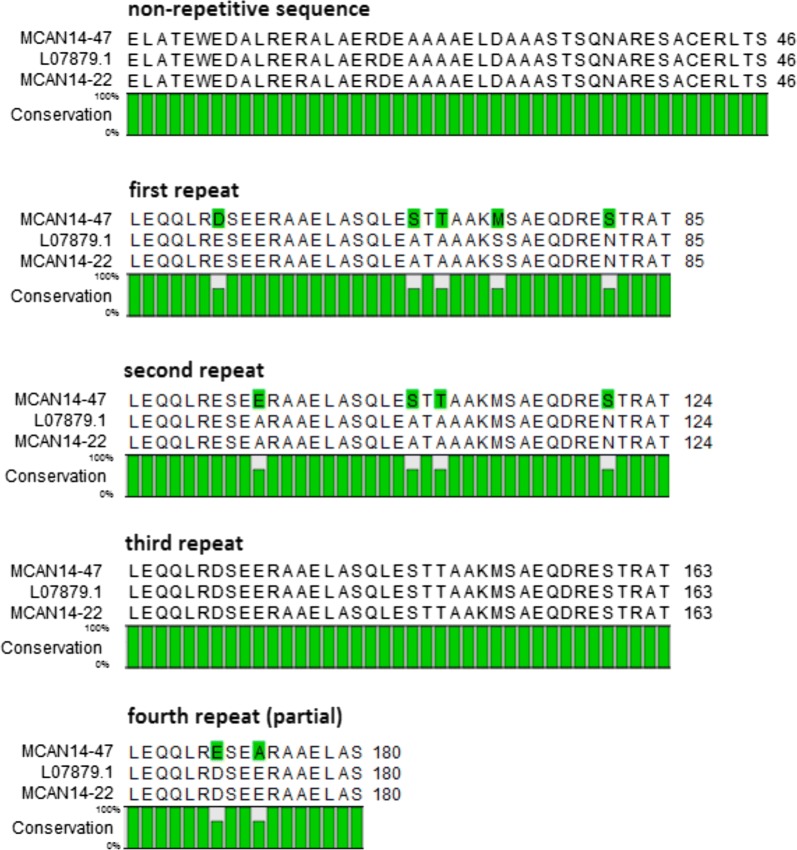

## Background

Visceral leishmaniasis (VL) or kala-azar is a neglected parasitic disease with an annual worldwide incidence of 500,000 human cases, caused by an obligate intracellular parasite belonging to the *Leishmania donovani* complex [1–3]. Early diagnosis and intervention are critical for symptomatic patients since the disease is lethal if left untreated [4]. The definitive diagnosis of VL is based on the visualization of the parasite in Giemsa-stained smears, obtained from the spleen, liver, bone marrow or lymph node. The main problem with parasitological diagnosis is its variable sensitivity and invasiveness [5, 6]. During a VL infection, substantial amounts of specific antibodies are produced that are not protective but can serve as a marker of ongoing or past infection with *Leishmania* sp. Accordingly, different serological tests such as immunofluorescence assay (IFAT), direct agglutination test (DAT), and enzyme-linked immunosorbent assay (ELISA) have been developed. Problems with IFAT, DAT and some ELISAs that use whole *Leishmania* parasites or crude extracts are batch to batch variation, the need for equipment and, most importantly, limited sensitivity [7–10]. Previous meta-analysis reported a sensitivity of 88% for IFAT, 87% for ELISA and 94% for DAT [11]. However, a study conducted in Iran, the region of the present study, reported a sensitivity of 70.5% for DAT [12].

The development of the recombinant antigen rK39 by Burns et al. [13] significantly contributed to the improvement of VL diagnosis. rK39 is a protein containing 39 amino acid repeats derived from a conserved region within a gene coding for a kinesin-related protein of *L. infantum* (former *L. chagasi*). Either using ELISA or simple immunochromatographic tests, high levels of antibodies against K39 are detectable in the blood of VL patients but not in those with mucosal or cutaneous leishmaniasis [10, 14–16]. Compared to other serological tests and test formats, rapid diagnostic tests (RDT) based on rK39 have the advantages of being fast, simple without the need for equipment and visual reading of the result [8, 10]. Nonetheless, rK39 RDTs show varying diagnostic performance in different parts of the world and even within a single region if heterogeneous *Leishmania* parasite populations are present [17]. Meanwhile, polymorphisms of the kinesin-related gene in various strains of *L. donovani* in different regions of the world may explain the discrepancies in sensitivity of the rK39 antigen in different serodiagnostic tests [18, 19].

Recombinant protein expression systems have been developed in prokaryotic organisms like bacteria and in eukaryotic cells and organisms such as yeast, mammalian, insect and plant cells, and protozoa like *L. tarentolae*, yet all have their advantages and drawbacks [20]. A major shortcoming of heterologous recombinant expression is codon bias and the processing of post-translational modifications [21]. *Leishmania tarentolae*, a trypanosomatid protozoan parasite of the Moorish gecko *Tarentola mauritanica*, was genetically engineered as a eukaryotic expression system (*Leishmania* expression system, LEXSY) for the production of recombinant proteins [22]. So far, rK39 and rK39-like antigens have been produced in heterologous expression systems [13, 23, 24]. We hypothesized that using *L. tarentolae* for expression of rK39 antigens derived from endemic *Leishmania* species would be advantageous in terms of codon usage and post-translational processing of the recombinant protein. Thus, the present study was undertaken to develop an alternative rK39 (*Li-*rK39), derived from an Iranian *L. infantum* strain and expressed in *L. tarentolae*, for serological diagnosis of VL in endemic regions of Iran.

## Methods

### Specimen collection

A total of 383 sera from 100 VL patients, 190 endemic healthy controls, 46 non-endemic healthy controls and 47 non-VL patients were included. Five ml of venous blood were collected in plain tubes to prepare serum. VL patients consisted of 48 male and 52 female children of 1 month to 16 years-old, all referred from VL-endemic regions and admitted to Nemazee Hospital, Shiraz University of Medical Sciences, Shiraz, Iran. All patients had ≥ 14 days’ fever, 88 had hepatosplenomegaly and 92 had anaemia (Hb < 11g/l). They were all positive in IFAT (titer > 64). After treatment with either antimonial or amphotericin B therapy, they were all cured with decreased spleen size. Endemic healthy controls were 190 children (55% male and 45% female) with age range of 1–16 years from endemic areas for VL in southern Iran, with no clinical symptoms and no history of VL. Non-endemic healthy controls included 46 individuals (50% male and 50% female) with an age range of 15–35 years, originating from non-endemic regions. Non-VL patients (*n* = 47) suffered from other infections: toxoplasmosis (*n* = 10); malaria (*n* = 10); cutaneous leishmaniasis (*n* = 11); fascioliasis (*n* = 7); and hydatidosis (*n* = 9). All sera from the endemic healthy controls, non-endemic healthy controls and non-VL patients were negative in IFAT (titer < 64).

### rK39 rapid diagnostic test

The test was conducted according to the manufacturer’s instructions (Kalazar Detect™ Rapid Test for VL, InBios, Seattle, USA).

### *Leishmania infantum* strain, DNA isolation and PCR amplification

A *L. infantum* strain (MCAN/IR/14/M14) was isolated from a domestic dog in Meshkin-Shahr area from north-western Iran in 2015 [25]. Promastigotes were cultured in 10 ml of HOMEM medium (GE Healthcare) supplemented with 10% heat-inactivated fetal calf serum and incubated at 26 °C. Pellets of 10^9^ *L. infantum* promastigotes were washed and suspended in PBS and genomic DNA was extracted using QIAamp Mini Kit (Qiagen, Hilden, Germany), according to the manufacturer’s instructions. DNA quantity was measured using NanoDrop spectrophotometer (NanoDrop ND-1000 UV-Vis spectrophotometer, NanoDrop Technologies, Wilmington, DE, USA).

### Construction of the recombinant expression vector

An analogous fragment of the K39 gene, as described by Burns et al. [13], was amplified from the Iranian *L. infantum* strain, using a forward primer selected to bind to the upstream non-repetitive part of GenBank sequence L07879, and a reverse primer able to target within most of the repetitive 117 bp regions (Table 1) [13]. As required for In-Fusion cloning, each primer was modified at the 5’-end by addition of a 15-bp sequence including a restriction site complementary to the place of integration in the expression vector, pLEXSY-hyg2 (Jena Bioscience, Jena, Germany). Using XbaI-rK39 F and KpnI-rK39 R primers, integration in pLEXSY-hyg2 provided a *L. mexicana* signal peptide at the N-terminal and added a hexa histidine-tag at the C-terminal, thus allowing IMAC purification. For cytoplasmic expression in pLEXSY-hyg2, the M14/47 sequence was re-cloned from pLEXSY-hyg2-M14/47 using NcoI-rK39 F and KpnI-rK39 R primers. This reconfiguration to pLEXSY-hyg2-CYTO-M14/47 cleaved off the existing signal peptide at the N-terminal, and provided a *Nco*I start codon. PCR reactions were performed in a final volume of 20 µl including 4 µl of 5× Phusion buffer (NEB), 200 µM of dNTPs (Eurogentec, Seraing, Belgium), 0.5 µM of each primer, 0.5 U of Phusion DNA polymerase (NEB), 4 µl of Q-solution 5× (Qiagen) and 10 ng genomic DNA. The final PCR program was run as follows: 1 cycle of 98 °C for 10 s; 35 cycles of 98 °C for 10 s, 67 °C for 30 s, 72 °C for 1 min; 1 cycle of 72 °C for 5 min. PCR products were separated on 1.5% agarose gels by electrophoresis and visualized, using ethidium bromide staining.

**Table 1:** Primers used in the PCR

Primer	Configuration	Sequence (5′–3′)
XbaI-rK39 F	Secreted	*CTGGCGCCTC***TCTAGA**GCTCGCAACC
NcoI-rK39 F	Cytoplasmic	*ACCAGATCTG***CCATGG**TCGCAACCGAGTGGGAGGA
KpnI-rK39 R	Hexa-histag	*TGGTGATGGTGGTG***GGTACC**ACTCGCCAGCTCC

*Notes*: Nucleotides in italics, sequence complementary to pLEXSY-hyg2; nucleotides in bold, restriction site; nucleotides underlined, K39 sequence from GenBank (L07879)

PCR products were purified, using the NucleoSpin PCR Clean-Up Kit (Macherey-Nagel, GmbH & Co. KG, Germany), according to the manufacturer’s instructions. Next, the purified amplicons were integrated into a *Xba*I or *Nco*I and *Kpn*I double digested pLEXSY-hyg2 vector, using In-Fusion recombinase. Finally, the completed vector was transformed into Stellar™ Competent Cells (Takara Bio Company, CA, USA) by heat shock and plated on Luria Bertani (LB) agar supplemented with 100 µg/ml carbenicillin at 37 °C overnight. At least 48 isolated *Escherichia coli* colonies were screened using colony PCR with primers that surround the site of integration in the pLEXSY vector (P1442 and A264). Using gel electrophoresis, all colonies that contained inserts larger than 300 bp were selected for further subculture in 5 ml LB medium overnight. Plasmids were harvested using the QIAprep Spin Miniprep (Qiagen) and sent for bi-directional sequencing (VIB Genetic Sequencing Facility, Antwerp, Belgium) using the P1442 and A246 primers. Next, translated protein sequences of the K39 gene were analysed by MUSCLE alignment with GenBank: L07879 reference sequence in CLC Sequence Viewer (Qiagen).

### Cultivation and transfection of *L. tarentolae*

*Leishmania tarentolae* strain P10 was cultivated in brain heart infusion (BHI) medium supplemented with hemin and penicillin-streptomycin. pLEXSY-hyg2-CYTO-M14/47, was linearized by *Swa*I digestion and concentrated to 330 ng/µl by QIAquick PCR purification kit (Qiagen). Transfections were performed with 10 µg of linearized plasmid *via* electroporation using the high voltage protocol on freshly passaged promastigotes, according to the Jena Bioscience manual [22]. Transgenic LEXSY strains were generated by polyclonal selection in BHI suspension culture, using 100 µg/ml hygromycin, after they were maintained under hygromycin pressure.

### Purification and characterization of recombinant protein

Production of recombinant protein *Li-*rK39 was carried out in 500 ml BHI medium, supplemented with 1.5% yeast extract [26], 2 g/l glucose [27], 100 µg/ml hygromycin and 0.2% hemin. After 72 h, when the OD_600_ reached 4, the cells were harvested by centrifugation for 10 min at 7500×*g* at 4 °C. The pellet was re-suspended in 50 ml of binding buffer (20 mM phosphate buffer pH 7.4, 500 mM NaCl, 20 mM imidazole), supplemented with 3 tablets of a protease inhibitor cocktail (Cat No. 05892791001, Roche Diagnostics, Mannheim, Germany), and lysed by five freeze-thaw cycles using liquid nitrogen, followed by 30 s sonication. After centrifugation for 30 min at 15,000×*g* at 4 °C, the supernatant was applied to an equilibrated 5 ml Ni-NTA agarose column. Bound proteins were eluted using 6 ml elution buffer (20 mM phosphate buffer pH 7.4, 500 mM NaCl, and 500 mM imidazole). Protein fractions were separated on 15% polyacrylamide gels under reducing conditions and stained with Coomassie brilliant blue G 250 (Merck, Darmstadt, Germany) or transferred onto a 0.45 μm nitrocellulose membrane (Bio-Rad), blocked with 5% non-fat dry milk in tris buffered saline (TBS, 0.1 M Tris-HCl, pH 7.5, 2.5 M NaCl), followed by incubation with anti-his-tag alkaline phosphatase conjugate (Cat No. 1396A, Bio-Rad, CA, US). The membrane was washed with TBS, containing 0.05% Tween-20 and incubated with substrate solution and developer (0.1 M Tris, pH 9.5, 0.1 M NaCl, and 5 mM MgCl_2_, NBT and BCIP). The reaction was stopped with H_2_O.

### ELISA

Microplates (Maxi binding, SPL Life Sciences, Eumhyeon, South Korea) were coated with 100 µl/well of purified *Li-*rK39 at a concentration of 0.5 μg/ml in 0.1 M carbonate/bicarbonate buffer (pH 9.6) overnight at 4 °C. Blocking was performed for 1 h at ambient temperature with 400 μl of PBS-Blotto (0.01 M phosphate, 0.14 M NaCl, pH 7.4). Diluted serum (100 μl of 1:100 dilution in PBS-Blotto) was added to each well and incubated for 30 min at ambient temperature. After three times washing with 350 µl/well of PBS-Tween (0.01 M, pH 7.4), 100 μl of horseradish peroxidase-conjugated goat anti-human IgG (Cat No. 109-035-003, Jackson ImmunoResearch, West Grove, PA, USA), diluted 1:40,000 in PBS-Tween (0.01 M, pH 7.4), were applied to the wells and incubated for 30 min at ambient temperature. After five times washing with 350 µl/well of PBS-Tween (0.01 M, pH 7.4), 100 µl/well of TMB substrate solution (Cat No. 34029, Thermo Scientific, Rockford, IL, USA) were added. The reaction was stopped with 100 µl/well of 1 N H_2_SO_4_. The absorbance (optical density, OD) at 450 nm and 630 nm was measured with a microplate reader (Epoch, Biotek Instruments, Winooski, VT, USA). The cut-off value for positive was defined as the OD corresponding with the highest value of the Youden index (*J* = sensitivity + specificity − 1).

### Statistical analysis

Sensitivity and specificity, with 95% confidence intervals (CI), of the rK39-ICT (Kalazar Detect™ Rapid Test for VL, InBios) were calculated using IBM® SPSS version 22 (IBM® SPSS Statistics, NY, USA).

Sensitivity and specificity at different cut-off points were calculated for the *Li-*rK39-ELISA and used to construct a receiver-operator characteristic (ROC) curve and to calculate the area under the curve (AUC) with its 95% CI.

Concordances between *Li-*rK39-ELISA and rK39-ICT, between *Li-*rK39-ELISA and IFAT, between rK39-ICT and IFAT were determined by calculating Cohen’s Kappa (*k*) and interpreted as follows: negligible (*k* = 0–0.20); weak (*k* = 0.21–0.40); moderate (*k* = 0.41–0.60); good (*k* = 0.61–0.80); and excellent (*k* = 0.81–1) [28, 29].

## Results

### Amplification and sequencing of the *L. infantum* K39 gene fragment

**Fig. 1:**
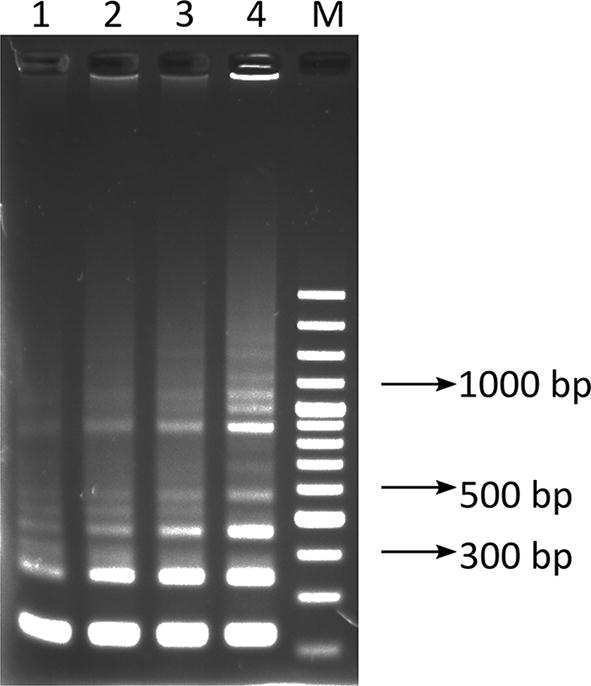
PCR amplified kinesin-like gene fragments from promastigotes of the Iranian *L. infantum* isolate MCAN/IR/14/M14 using XbaI-rK39F and KpnI-rK39R primers. Lanes 1–4: PCR products generated at annealing temperature of 64 °C, 65 °C, 66 °C and 67 °C, respectively; Lane M: GeneRuler 100 bp Plus DNA (Thermo Fisher Scientific)

PCR amplification of the *L. infantum* K39 fragment using the XbaI-K39F and KpnI-K39R primers resulted in a multi-banded amplification product, which could not be reduced to single bands by varying the annealing temperature (Fig. 1) or by using a different cloning polymerase. Therefore, PCR products were co-purified and used for cloning in the pLEXSY-hyg2 expression vector. Individual recombinant clones screened by colony PCR revealed in each the integration of a single fragment; however, between the different clones these fragments had various sizes, usually differing in 117 bp from each other, corresponding with the size of one 39 aa repeat. In total, six plasmids yielded rK39 sequences from MCAN/IR/14/M14 with a length greater than 300 bp, for which the largest, MCAN14/47 and MCAN14/22 had a size of 550 bp. Sequences of these two genes were deposited in the GenBank database with accession numbers MN585730 (MCAN14/47) and MN585731 (MCAN14/22).

**Fig. 2:**
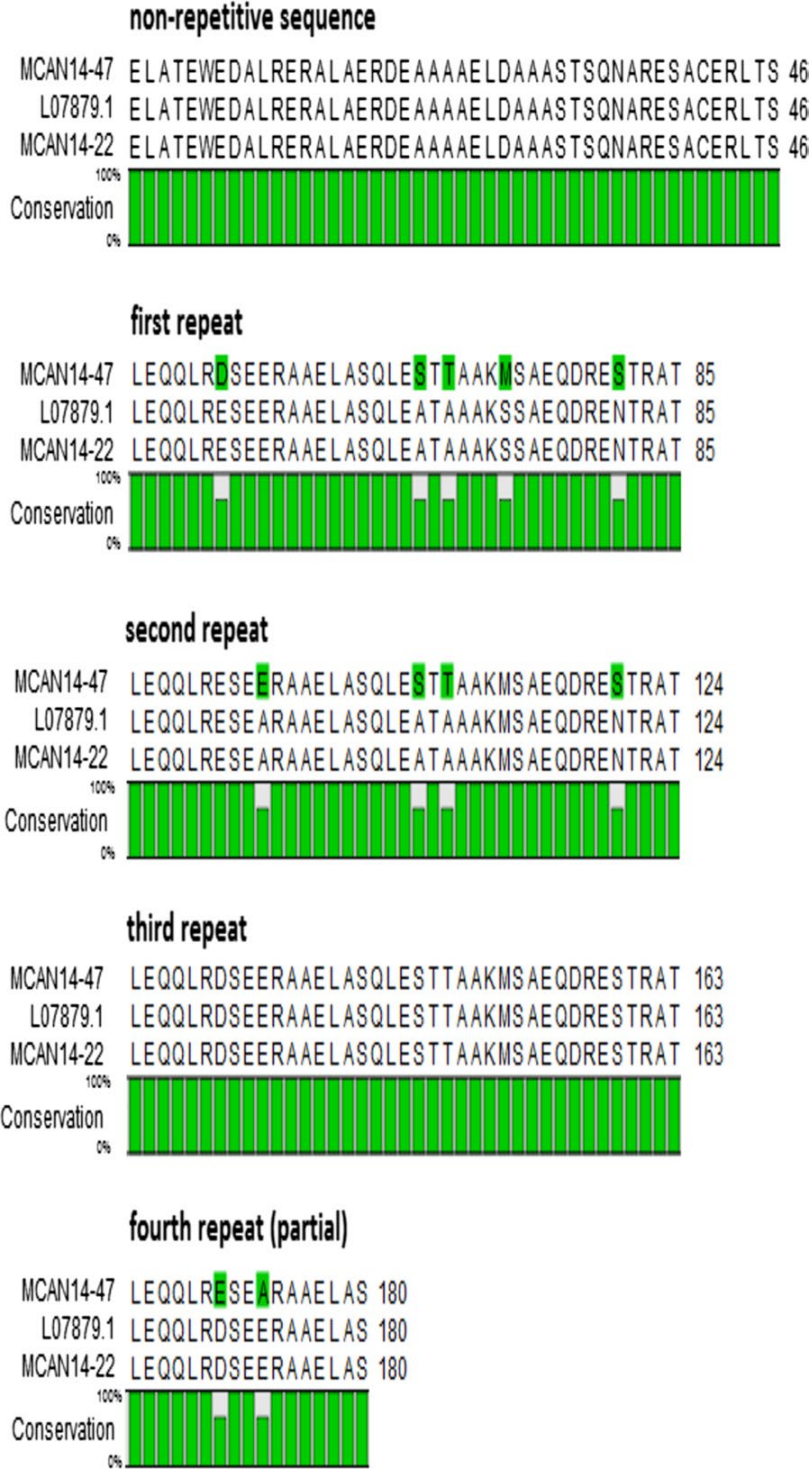
Amino acid alignment of the corresponding fragment of the translated GenBank reference sequence L07879 [13] with the two longest sequences from K39 clones from MCAN/IR/14/M14 obtained in pLEXSY-hyg2: MCAN14/47 (GenBank: MN585730) and MCAN14-22 (GenBank: MN585731)

When translated to protein, the sequences revealed that the Iranian *L. infantum* K39 appears to have at least 2 alleles. The first allele, from clone MCAN 14/22, had 100% identity at 180 amino acids positions of the corresponding translated *L. chagasi* kinesin-like gene (GenBank: L07879). In contrast, the second allele, from clone MCAN14/47 had no substitutions in the N-terminal non-repetitive part, 5 amino acids substitutions in the first repeat, 4 substitutions in the second repeat, none in the third repeat and 2 substitutions in a partial fourth repeat (Fig. 2), thus, resulting in only 94% protein similarity to the corresponding fragment from GenBank (L07879). The MCAN14/47 from pLEXSY-hyg2 was re-cloned using NcoI-K39 F and KpnI-K39 R primers into pLEXSY-hyg2-CYTO-MCAN14/47. Here, the artificial start codon provided by *Nco*I substituted the first amino acid, E, and the second, L, from MCAN14/47 to M and V, respectively in order to allow cytoplasmic expression of the 188 amino acid long *Li-rK39* construct.

### Expression and purification

**Fig. 3:**
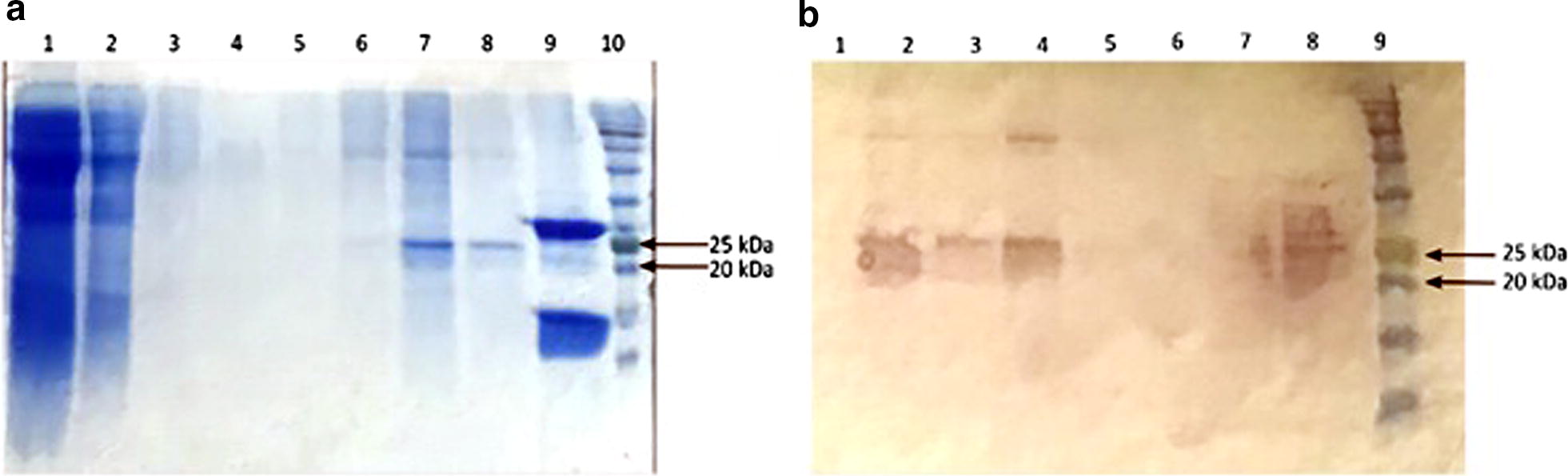
SDS-PAGE (**a**) and Western blot (**b**) showing the protein composition of the different fractions collected from *L. tarentolae* clone pLEXSY-hyg2-MCAN14/47, before and after the purification on the Ni-NTA. **a** Lane 1: whole cell lysate; Lane 2: flow-through from Ni-NTA column; Lanes 3 and 4: wash fractions; Lanes 5–8: elution fractions; Lane 9: recombinant His-tagged H2B (positive control); Lane 10: 11–245 kDa protein marker. **b** Lanes 1–6: elution fractions; Lane 7: flow-through; Lane 8: whole cell lysate; Lane 9: 11–245 kDa protein marker

The pLEXSY-hyg2-MCAN14/47 plasmid was transfected to *L. tarentolae* and polyclonal selection using hygromycin yielded a recombinant population. This recombinant population expressed a cytosolic recombinant protein, *Li-*rK39, which could be purified on Ni-NTA resin. The yield of this purified protein was 250 µg/ml in 500 ml culture. The presence of the recombinant *Li-*rK39 protein and its purity was confirmed by SDS-PAGE and Coomassie blue staining (Fig. 3a) and by Western blot, using an anti-his-tag antibody (Fig. 3b). The apparent size of the protein was 25 kDa although the expected size was 21 kDa (including the 6 histidine residues), probably due to post-translational modification like glycosylation or due to the relatively high content in basic amino acids (34%).

### Diagnostic performance of *Li-*rK39 in an ELISA

**Fig. 4:**
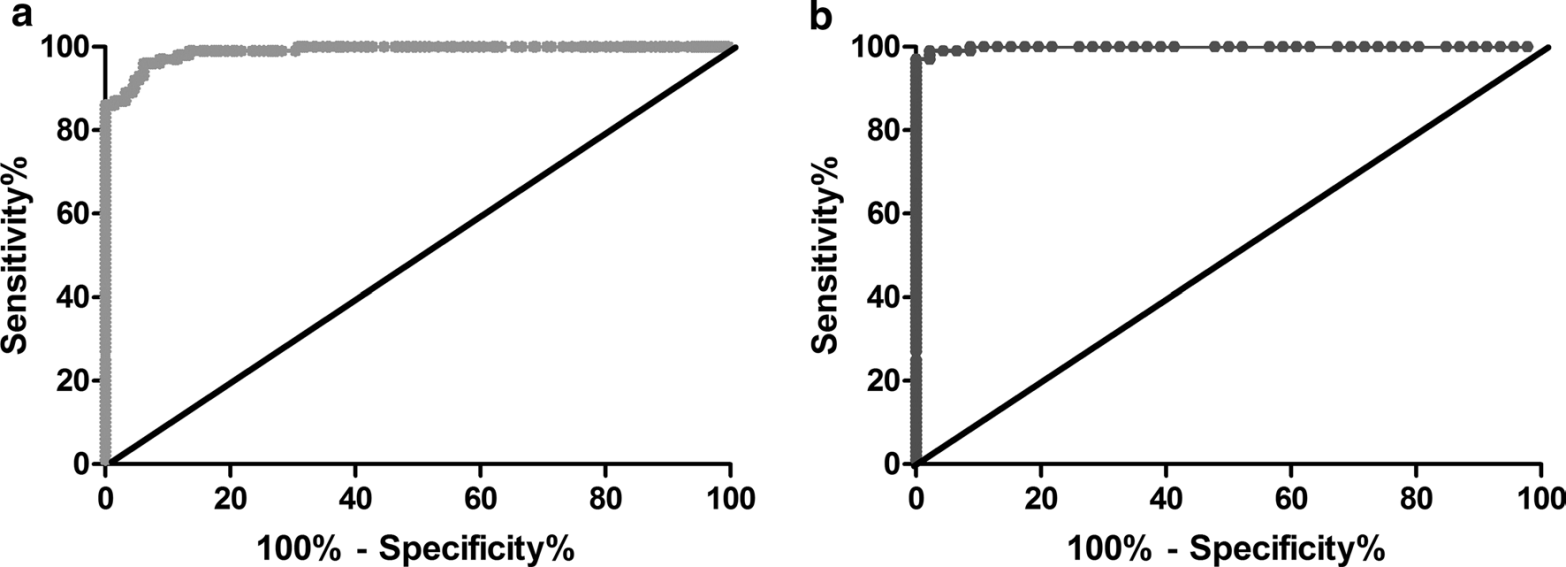
Receiver operator characteristic curves constructed from ELISA results. **a** ROC curve obtained with sera from VL patients and all the samples. **b** ROC curve obtained with sera from VL patients and non-endemic controls

The diagnostic performance of the *Li-*rK39 antigen was assessed in an ELISA with sera from 100 VL patients, 190 endemic healthy controls, 46 non-endemic healthy controls and 47 non-VL patients. With IFAT as reference test and the cut-off OD of 0.312, corresponding with the highest Youden index (*J* = sensitivity +specificity − 1). The *Li-*rK39 antigen showed a sensitivity of 96.0% (95% CI: 90.1–98.9%) and a specificity of 93.8% (95% CI: 90.3–96.4%) yielding an AUC of 0.989 (95% CI: 0.981–0.997) by using endemic, non-endemic healthy controls and non-VL patients (Fig. 4a). Sensitivity and specificity using only non-endemic controls at cut-off OD of 0.295 was 97% (95% CI: 91.5–99.4%) and 100% (95% CI: 93–100%) yielding an AUC of 0.999 (95% CI: 0.996–1) (Fig. 4b). Two of the non-VL patients, including malaria and cutaneous leishmaniasis showed a positive reaction in the *Li*-rK39 ELISA.

### rK39 ICT (InBios) results

With IFAT as reference test, the rK39 ICT exhibited a sensitivity of 90.0% (95% CI: 82.4–95.1%) and a specificity of 100% (95% CI: 98.5–100%).

### Concordance between different tests

By including all the samples including endemic, non-endemic and non-VL patients, the concordance between the *Li-*rK39 ELISA and the rK39-ICT was good with *k* = 0.80 and *P* < 0.0001. Concordance between the *Li*-rK39 ELISA and IFAT and between rK39-ICT and IFAT was excellent with *k* = 0.86 and 0.92, respectively, and *P* < 0.0001.

## Discussion

Given the fact that VL has a fatal outcome if not treated, its early and accurate diagnosis is necessary. Immunochromatographic and ELISA tests, based on a recombinant antigen containing 39 amino acid repeats of a kinesin-related protein of a Brazilian *L. infantum* strain, are commercially available. Previous studies showed a considerable variation in the sensitivity and specificity of such tests, depending on test format and the geographical distribution of VL with reported sensitivities between 67.6–100% and specificities between 59–100% for rK39-ICTs. The rK39-ELISA has been shown to have sensitivity and specificity ranges between 88.6–100% and 84.5–100%, respectively [17]. A prospective study conducted in Iran on 17 VL patients and 137 patients with other diseases reported a sensitivity of 82.4% and specificity of 100% for the InBios rK39-ICT [28]. Such results imply that due to the suboptimal diagnostic accuracy of rK39-based tests, other diagnostic tests such as IFAT or quantitative PCR need to be performed, which are time-consuming, require equipment and well-trained personnel.

Bhattacharyya et al. [19] illustrated an extensive diversity of K39 among various strains of *L. donovani* which could affect the accuracy of rK39-ICTs. Indeed, in the present study, we confirm that there is also diversity in the K39 gene within an Iranian *L. infantum* isolate. Cloned 550-bp fragments of the K39 gene from the Iranian isolate MCAN/IR/14/M14 showed that this locus is heterozygous, carrying a sequence identical to the one described for Brazilian *L. infantum*, and also a variant that differed by 6% with the amino acid sequence of the Brazilian K39 sequence. Surprisingly, these amino acid substitutions were mostly found at the same positions to those noted in the *L. donovani* study by Bhattacharyya et al. [19], yet, not all substitutions were same as the ones in *L. donovani*. The observed difference in protein sequence within this limited range (3.5 repeats) might already affect the performance of ICTs. Therefore, we chose this variant, called *Li*-rK39, for expression in *L. tarentolae*.

In the present study, the ELISA with the purified recombinant *Li*-rK39 fragment was more sensitive (96%). One of the four false negative serum samples in the ELISA was also negative in ICT. The reason that some IFAT-positive VL patients remained negative in the *Li*-rK39 ELISA is not clear. Elfadi et al. [18] pointed out that it is hard to select a diagnostic test based on an antigen of a single strain of *Leishmania* in places where a population of heterogeneous parasites exists. This is illustrated by a study conducted in India, where a sensitivity of 100% was reported for an ELISA using an rK39 derived from an Indian isolate of *L. donovani* KE16 [24]. It may therefore be interesting to investigate the heterogeneity within the kinesin-related K39 gene of *L. infantum* isolated from a human patient in a given area, and to express the dominant sequence as a recombinant antigen for diagnostic purposes. Furthermore, it should be kept in mind that some cases of VL in Iran are caused by *L. tropica* [29, 30].

A study previously conducted in Iran on 12 parasitologically confirmed VL patients reported a sensitivity of 91% for ELISA with a recombinant antigen consisting of one 39 amino acid repeat of the K39 of an Iranian *L. infantum* strain, expressed in *E. coli* [31]. Compared to our study, the low sensitivity obtained in their study may be due to the use of only one repeat unit of K39, derived from a different *L. infantum* isolate and expressed in a prokaryotic host.

Another study showed that a higher copy number of K39 repeats in a recombinant antigen derived from *L. infantum* increased the affinity of antibodies to the antigen [32]. In the present study, the sensitivity of *Li-*rK39-ELISA is greater than the one conducted in Brazil and equal to the one in India which used a commercial rK39 of *L. infantum* in their ELISA system [24, 33]. Also, the sensitivity of the *Li-*rK39-ELISA was greater than that of rK39-ICT (InBios) (90%). This might be due to the higher analytical sensitivity of the ELISA compared to the ICT format. However, in contrast, we found three VL patients positive in the rK39-ICT that were negative in the Li-rK39-ELISA. This discordance may be due to: (i) small modifications of the original sequence by adding an N-terminal methionine for cytoplasmic expression; (ii) the different solid phases in ELISA (polystyrene) and ICT (nitrocellulose) that may influence the antigen conformation; (iii) the more stringent antibody-antigen binding conditions in the ELISA; and (iv) the fact that we expressed and evaluated only one of the two alleles of the kinesin related protein gene, which is different from the one used to produce the Brazilian *L. infantum* rK39 in the rK39-ICT. It follows that combining in one test recombinant proteins derived from both alleles may further increase the sensitivity of the ELISA.

The *Li-*rK39-ELISA revealed a specificity of 100% with the 46 non-endemic controls but 14 of the 190 endemic controls and two of the non-VL patients including cutaneous leishmaniasis and malaria were positive, although with OD below 0.5, resulting in a specificity of 93.8% which is not unexpected since in VL endemic regions a considerable number of infected people remain asymptomatic but develop a detectable antibody response [34].

In the present study, we used the *L. tarentolae* expression system (pLEXSY) to produce *Li*-rK39 and have evaluated its diagnostic performance. So far, the rK39 antigen has been produced in prokaryotic expression systems. Post-translational modification can affect an antigen in many aspects like stability, solubility, resistance against proteases etc. but doesn’t occur in prokaryotic expression systems. Also, codon bias is an important issue related to expression systems [21, 35]. The main advantage of prokaryotic expression systems is their low cost, easy handling and high yield [21, 35]. *Leishmania tarentolae* is a relatively easy eukaryotic expression system but compared to bacteria, it has a lower growth rate (doubling time of 6 h in agitated culture) and lower yield. On the other hand, it can be considered as a suitable expression system for rK39 of *L. infantum*, so the recombinantly produced protein may be more similar to its native counterpart. All these factors make this expression system suitable for the expression of proteins of kinetoplastid organisms as Rooney et al. [36] have shown in their study.

## Conclusions

Considering the diversity of the kinesin-related gene encoding rK39 among the different strains of *Leishmania* species in different regions of the world, it can be suggested that the recombinant antigens be generated based on the dominant parasite strains circulating in a given region. Derived from the rK39 sequences found in an Iranian *L. infantum* strain isolated from a dog, a recombinant antigen showed satisfactory diagnostic accuracy when tested on sera from VL patients, endemic and non-endemic controls. Besides, we found that *L. tarentolae* is a good expression system for producing the rK39 antigen of *L. infantum*.

## Data Availability

Data supporting the conclusions of this article are included within the article. The raw data obtained during the present study are available upon request.

## Abbreviations

VL: visceral leishmaniasis
*Li*-rK39: *L. infantum*-rK39
ICT: immunochromatographic test
IFAT: immunofluorescence assay
DAT: direct agglutination test
ELISA: enzyme-linked immunosorbent assay
RDT: rapid diagnostic tests
BHI: brain heart infusion
TBS: tris-buffered saline
CI: confidence intervals
ROC: receiver-operator characteristic
AUC: area under the curve
*k*: kappa
